# Supplementary System of Nutrient Arteries for the Lungs

**Published:** 1865-02

**Authors:** 


*Supplementary System of Nutrient Arteries for the Lungs.*—At the last meeting of the British Scientific Association, Dr. Turner read a paper in relation to this subject. An arterial plexus was described on the side of the pericardium beneath the mediastinal pleura. It was formed by the junction of the pericardiac, mediastinal, and phrenic branches of the internal mammary artery with each other, and with numerous fine branches from the trunks of the intercostal arteries. From it a number of slender thread-like arteries passed to the lung, some in front of its root, others behind, and others between the layers of the ligamentum latum pulmonis. Some of these arteries were distributed in in the substance of the lung, others on its surface beneath the pulmonic pleura. Through the agency of this plexus an arterial communication ts established between the bloodvessels of the lung and the arteries which supply the wall of the chest with blood.

